# S1‐Leitlinie: Therapie der generalisierten pustulösen Psoriasis: S1 Guideline: Therapy of generalized pustular psoriasis

**DOI:** 10.1111/ddg.70244

**Published:** 2026-11-10

**Authors:** Rotraut Mößner, Neda Cramer, Sascha Gerdes, Ulrike Hüffmeier, Andreas Körber, Kai Krause, Christian Kromer, Katharina Antonia Langen, Sandra Philipp, Knut Schäkel, Michael P. Schön, Michael Schultheis, Lukas Sollfrank, Petra Staubach, Michael Sticherling, Johannes Wohlrab, Dagmar Wilsmann‐Theis, Cornelia Paul

**Affiliations:** ^1^ Klinik für Dermatologie Venerologie und Allergologie Universitätsmedizin Göttingen; ^2^ Zentrum für entzündliche Hauterkrankungen Klinik für Dermatologie Venerologie und Allergologie Universitätsklinikum Schleswig‐Holstein, Campus Kiel; ^3^ Institute of Human Genetics Centre for Personalized Medicine and Research (CESAR) Universitätsklinikum Erlangen Friedrich‐Alexander‐Universität Erlangen‐Nürnberg; ^4^ Hautärzte RÜ 143, Essen; ^5^ Universitätsklinik und Poliklinik für Dermatologie und Venerologie Martin‐Luther‐Universität Halle‐Wittenberg; ^6^ Hautarztpraxis Dres. med. M. Friedrich/S. Philipp, Oranienburg; ^7^ Hautklinik Universitätsklinikum Heidelberg; ^8^ Hautklinik und Poliklinik der Universitätsmedizin Mainz Johannes Gutenberg‐Universität KöR, Mainz; ^9^ Hautklinik Erlangen Deutsches Zentrum Immuntherapie (DZI) Uniklinikum Erlangen; ^10^ Zentrum für Hauterkrankungen Klinik für Dermatologie und Allergologie Universitätsklinikum Bonn; ^11^ Institut für Klinische Chemie und Klinische Pharmakologie Universitätsklinikum Bonn

**Keywords:** Genetik, GPP, Leitlinie, Therapie, Genetics, GPP, guideline, therapy

## Abstract

Die S1‐Leitlinie „Therapie der generalisierten pustulösen Psoriasis (GPP)“ ist eine deutsche Leitlinie, die nach den Kriterien der AWMF erstellt wurde. In der Langfassung werden Aspekte wie klinisches Bild, Pathogenese, Diagnose und Differenzialdiagnose, Begleiterkrankungen und Therapien beleuchtet. Ferner wurden dort Therapieempfehlungen für Erwachsene mit GPP verabschiedet. Hierbei wurden sowohl zugelassene Therapien als auch Off‐Label‐Therapien berücksichtigt. Der vorliegende Artikel stellt eine gekürzte Fassung der Leitlinie dar, wobei die Therapieempfehlungen vollständig und ungekürzt aufgeführt werden.

## Summary

The S1 guideline “Therapy of generalized pustular psoriasis (GPP)” is a German guideline developed in accordance with the criteria of the AWMF. The full version addresses clinical presentation, pathogenesis, diagnosis and differential diagnosis, comorbidities, and therapy. In addition, therapy recommendations for adults with GPP are provided. Both approved and off‐label therapies are considered. The present article is a shortened version of the guideline, in which the therapy recommendations are presented in full and without omission.

## EINLEITUNG

In der vorliegenden Leitlinie werden allgemeine Aspekte zur generalisierten pustulösen Psoriasis, die Lokaltherapie, die physikalische Behandlung und die medikamentöse Systemtherapie von Erwachsenen behandelt. In Abbildung 1 sind die Empfehlungen zur Behandlung der generalisierten pustulösen Psoriasis vereinfacht dargestellt.

**ABBILDUNG 1:**
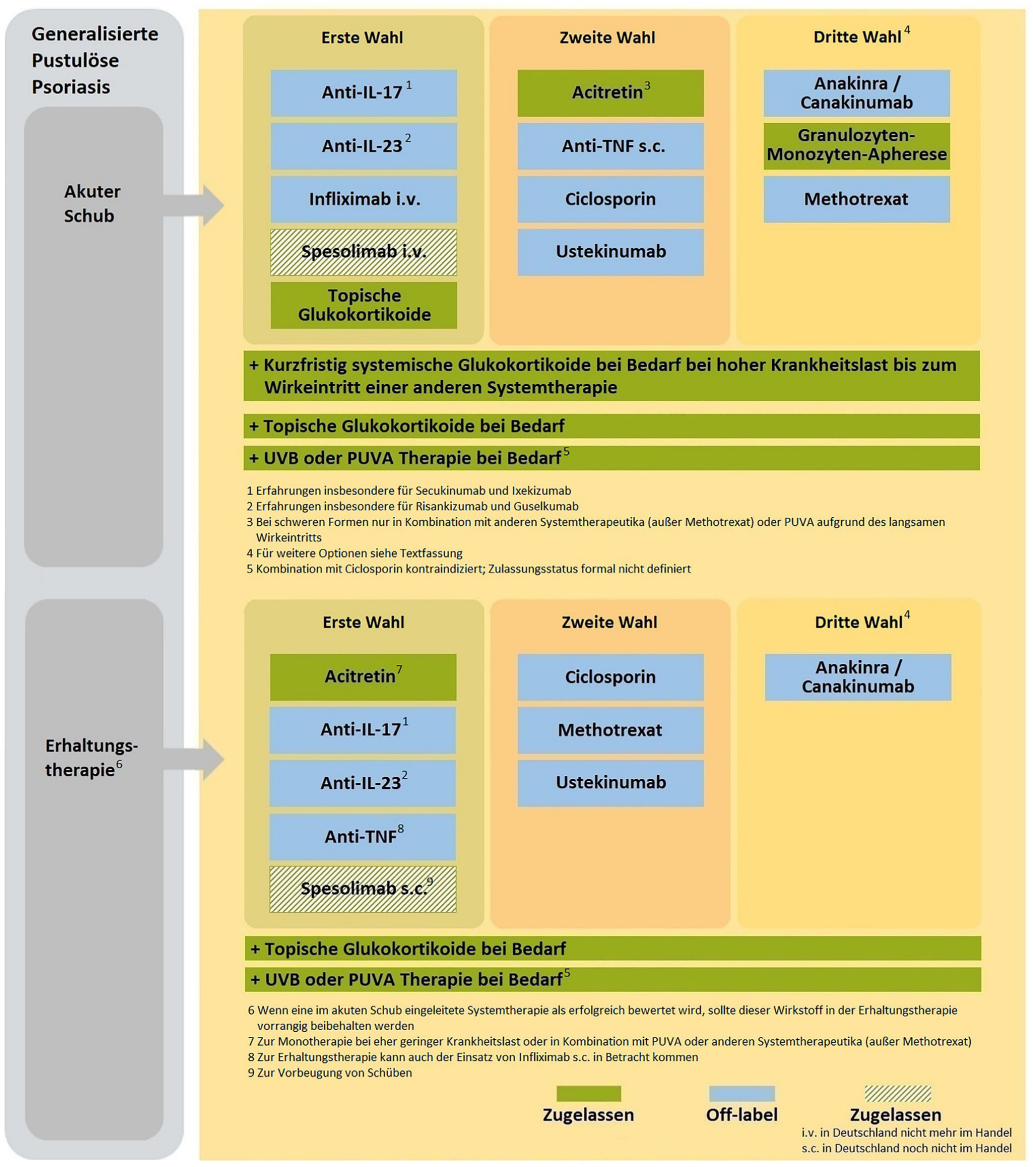
Vereinfachte Empfehlungen zur Behandlung der generalisierten pustulösen Psoriasis.

Die Therapieempfehlungen sind vollständig und ungekürzt enthalten, während die Inhalte zum medizinischen Hintergrund und verfügbaren Studien in gekürzter Form dargestellt werden. Die ungekürzte Langfassung der Leitlinie ist auf der Website der AWMF unter https://register.awmf.org/de/leitlinien/detail/013‐107 verfügbar.

### Definition

Die generalisierte pustulöse Psoriasis (GPP) ist eine seltene, schwere und potenziell lebensbedrohliche autoinflammatorisch vermittelte neutrophile Dermatose. Sie wurde als Form der pustulösen Psoriasis erstmalig 1909 durch von Zumbusch beschrieben.^1^

Das *European Rare and Severe Psoriasis Expert Network* (ERASPEN) definiert die GPP durch großflächige Hauterytheme mit sterilen, makroskopisch sichtbaren Pusteln, die entweder persistieren (> 3 Monate) oder schubhaft mit mindestens zwei Episoden sind. Die GPP kann sowohl mit als auch ohne systemische Entzündung und Minderung des Allgemeinbefindens auftreten, ebenso mit oder ohne Psoriasis vulgaris.^2^, ^3^, ^4^, ^5^

Unter einem akuten Schub wird eine deutliche Befundverschlechterung verstanden, oft einhergehend mit Beschwerden wie Schmerzen und/oder mit einer Reduktion des Allgemeinbefindens.

### Epidemiologie

Es liegen nur wenige Daten zur Epidemiologie der GPP vor. Die Prävalenz scheint je nach ethnischer Abstammung zu variieren und bei asiatischen Ethnien im Vergleich zur europäischen Bevölkerung deutlich erhöht zu sein. In Japan liegt sie laut Daten von 1996 bei 7,46 pro Million Einwohner,^6^ während in Frankreich 2004 bei der Befragung von 121 dermatologischen Kliniken eine Prävalenz von 1,76 pro Million erfasst wurde.^7^ Die Auswertung einer Datenbank der südkoreanischen Krankenversicherung von 2011 bis 2015 belegte eine deutlich höhere Prävalenz von 88 bis 124 Fällen pro Million Einwohner.^8^ Für Deutschland liegen keine absoluten Zahlen vor. In einem Übersichtsartikel zur GPP wurde anhand stationärer Behandlungsfälle der GPP in Deutschland im Jahr 2019 (395 stationäre Aufnahmen) und der Annahme, dass bei Patienten mit GPP im Schnitt alle 5 Jahren ein Schub auftrete, eine Prävalenz von 23,7 pro Million geschätzt.^9^ Da die GPP eine sehr seltene Krankheit mit hoher diagnostischer Unschärfe ist, müssen diese Zahlen kritisch gesehen werden. Sie entsprechen vermutlich nicht der epidemiologischen Realität. Einschätzungen nach sind etwa 80 % aller Psoriasisfälle der Psoriasis vulgaris zuzuordnen, 20 % den pustulösen Formen, aber nur 2 % der GPP, mit höherer Prävalenz bei Frauen.^5^, ^10^, ^11^, ^12^ Die meisten Erkrankungen beginnen zwischen dem 40. und dem 60. Lebensjahr,^11^ jedoch wurde auch das Auftreten im Kindesalter beschrieben.^13^

### Pathogenese

Der GPP liegt eine autoinflammatorische Entzündungsreaktion mit fehlerhafter Kontrolle des Interleukin (IL)‐36‐Signalweges und auch anderer Mediatoren zugrunde.^3^, ^9^ Vielfach lösen Infekte die unspezifische Immunabwehr und die initiale IL‐36‐Produktion aus. Das aktuelle Modell der Immunpathogenese ist in Abbildung 2 zusammengefasst und wird in der Legende beschrieben.

**ABBILDUNG 2:**
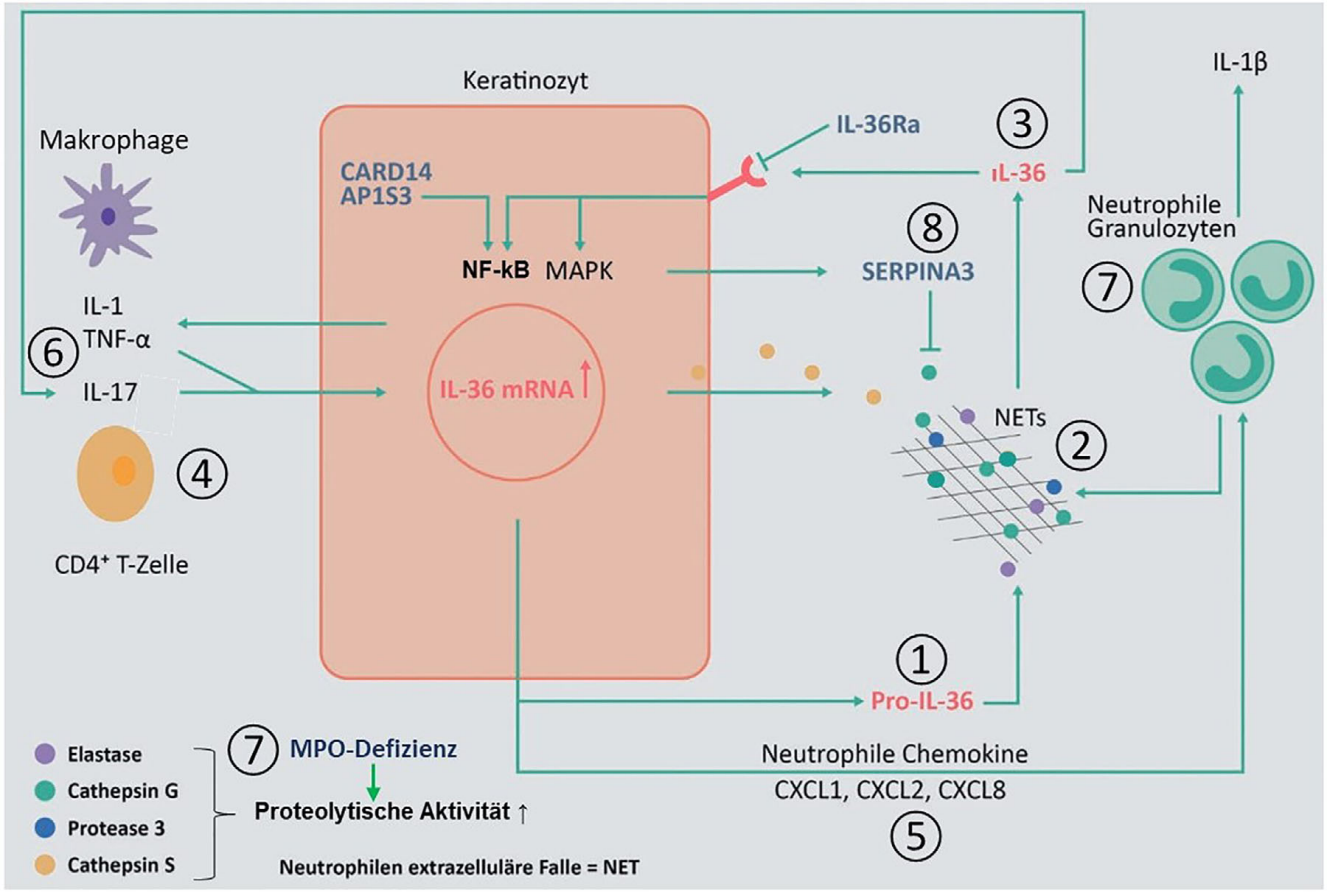
Pathogenese der GPP (modifiziert nach 9). IL‐36 wird von Keratinozyten als inaktives Vorläuferprotein (α, β oder γ) freigesetzt (pro‐IL‐36) ①. Seine biologische Aktivität erlangt es extrazellulär durch proteolytische Spaltung ②.^57^ Zu den IL‐36‐aktivierenden Proteasen gehört das überwiegend von mononukleären Phagozyten (Makrophagen und dendritische Zellen) produzierte Cathepsin S. Protease 3, Cathepsin G und Elastase sind weitere IL‐36‐aktivierende Proteasen, die besonders bei entzündlichen Reaktionen von neutrophilen Granulozyten zusammen mit den neutrophilen extrazellulären Fallen (neutrophil extracellular traps, NET) freigesetzt werden. IL‐36 stimuliert autokrin die proinflammatorische Aktivierung von Keratinozyten ③ und führt darüber hinaus zur Aktivierung von Endothelzellen, Fibroblasten und Immunzellen ④. Klinisches Kennzeichen ist das eruptive Auftreten von Pusteln, dem eine Expression von Chemokinen zur Rekrutierung neutrophiler Granulozyten zugrunde liegt ⑤. Zusätzlich sind T‐Zellen und Makrophagen an der Entzündung beteiligt ④. Zusammen prägen sie das lokale Zytokinmilieu mit Expression von IL‐36, IL‐1, TNF‐α und IL‐17A ⑥.^57^ Eine Myeloperoxidase (MPO)‐Defizienz korreliert mit der Aktivität von Serinproteasen ⑦ und führt unter anderem zu reduzierter NET‐Bildung ②. SERPINA3 hemmt die Serinprotease Cathepsin G ⑧. Proteine, die von Genen kodiert werden, bei denen seltene Varianten für pustulöse Formen der Psoriasis prädisponieren, sind in blau dargestellt. *Abk*.: IL‐36Ra, IL‐36‐Rezeptor‐Antagonist; IL‐1β, Interleukin‐1β; TNF‐α, Tumornekrosefaktor alpha; IL, Interleukin; CARD14, Caspase‐Rekrutierungsdomänen‐Familienmitglied 14; AP‐1, Adapterprotein‐Komplex 1; AP1S3, Komplex‐Untereinheit Sigma 3; SERPINA3, Serinprotease‐Inhibitor A3; NF‐κB, *nuclear factor kappa‐light‐chain‐enhancer of activated B cells*; MPO, Myeloperoxidase; MAPK, mitogenaktivierte Proteinkinase; CD4+, Cluster of Differentiation 4; NET, neutrophile extrazelluläre Falle; CXCL, C‐X‐C‐Motiv‐Chemokin‐Ligand

### Genetische Grundlagen

Siehe Langfassung der Leitlinie.

### Provokationsfaktoren

Die Provokationsfaktoren der generalisierten pustulösen Psoriasis sind in Tabelle 1 dargestellt.

**TABELLE 1:** Provokationsfaktoren der generalisierten pustulösen Psoriasis.

Medikamente Betablocker, Antimalariamittel, Lithium, Kontrazeptiva, Terbinafin, Glukokortikoide
Infektionen
Biologika (vor allem TNF‐Inhibitoren, selten Interleukin‐17‐ oder Interleukin‐23‐Blocker (im Sinne einer paradoxen Reaktion))
Starke UV‐Exposition
Schwangerschaft

### Klinisches Bild und Verlauf

Disseminierte Erytheme und Schwellungen sowie konfluierende sterile Pusteln kennzeichnen die GPP (Abbildung 3).^1^ Häufig ist das Allgemeinbefinden stark eingeschränkt und es kann zu Arthritiden kommen. Labordiagnostisch findet man oft Leukozytose mit Neutrophilie sowie deutlich erhöhte Werte von CRP und BSG, seltener Hypokalzämie und Hypoproteinämie.^11^, ^14^, ^15^, ^16^ Bei der GPP unterscheidet man den Krankheitsbeginn im Kindesalter (early onset) von dem im Erwachsenenalter (adult onset).^11^, ^17^, ^18^, ^19^, ^20^

**ABBILDUNG 3:**
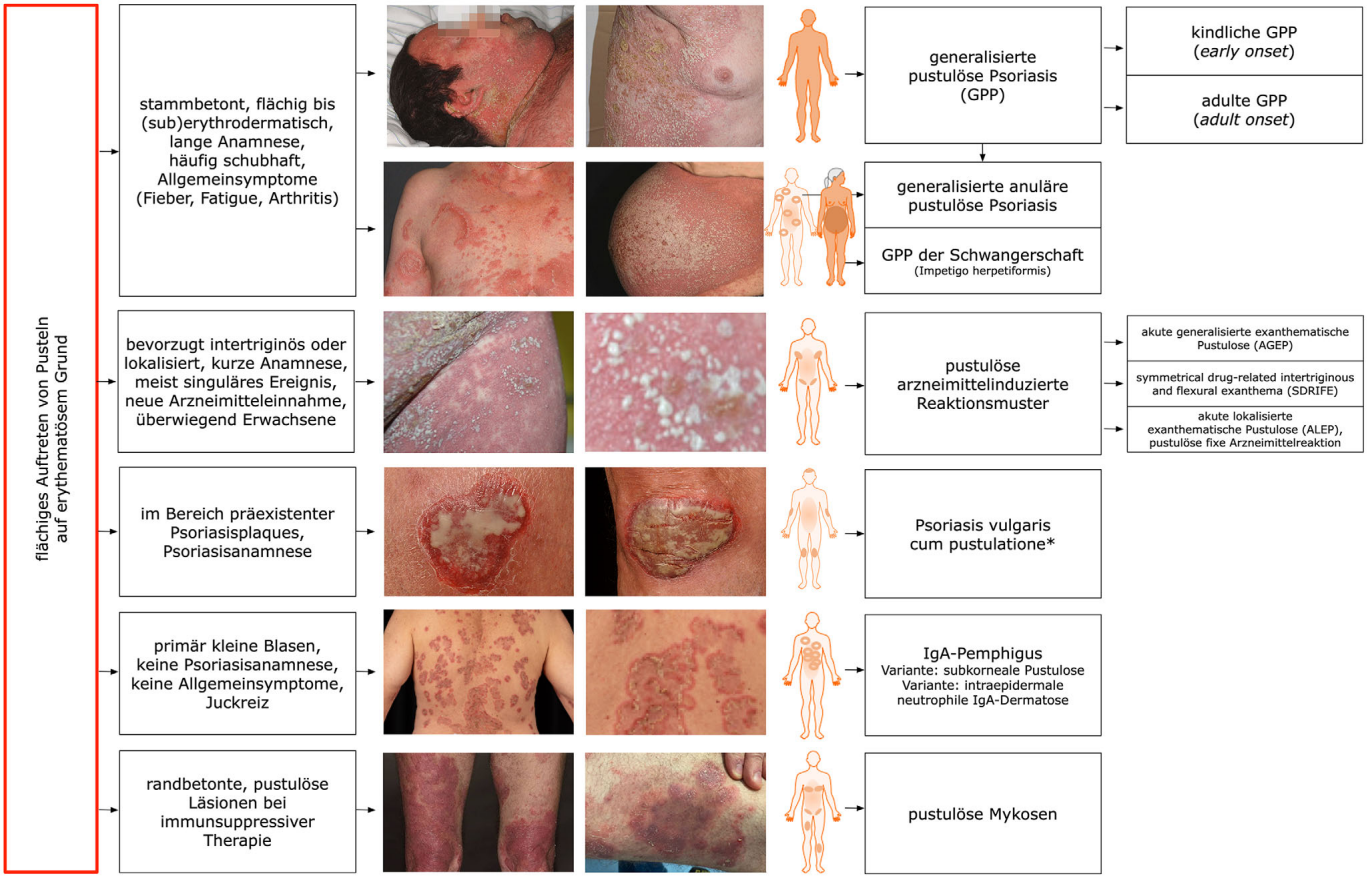
Differenzialdiagnostische Kriterien. *Quelle: Klinische Bilder von Herrn Prof. Dr. Ulrich Mrowietz zur Verfügung gestellt.

Histologische Kennzeichen der GPP sind auffällige Ansammlungen neutrophiler Granulozyten im Stratum spinosum (spongiforme Kogoj‐Pusteln) sowie im Stratum corneum (Munrosche Mikroabszesse). Andere Merkmale wie Parakeratose, Akanthose, Hyperkeratose, verlängerte Reteleisten, verschmälertes Stratum granulosum sowie erweiterte Kapillaren in den dermalen Papillen, wie sie auch typischerweise bei der Psoriasis vulgaris gesehen werden, sind manchmal ebenfalls nachweisbar, können bei akuten Verläufen oder in Frühphasen aber auch (noch) fehlen.^21^

### Komorbidität

Die Komorbidität der GPP wird maßgeblich beeinflusst von der systementzündlichen Last, die sowohl Krankheitsaktivität (Schweregrad) als auch Krankheitsdauer (Schubhäufigkeit und Schubdauer) beinhaltet. Sie schließt kardiovaskuläre Erkrankungen wie arterielle Hypertonie, ischämische Herzkrankheit, Schlaganfall, und metabolische Erkrankungen wie Hyperlipidämie und Diabetes mellitus ein.^17^, ^18^, ^19^ Darüber hinaus muss durch die erhebliche Einschränkung der Lebensqualität in Verbindung mit neuroinflammatorischen Vorgängen auch von einer besonderen psychischen Belastungssituation (Angststörung, Depression) ausgegangen werden.^14^, ^22^, ^23^, ^24^, ^25^

Zudem tritt die Lingua geographica als unspezifisches Zeichen der chronischen Entzündung gehäuft auf.^26^, ^27^ In einer retrospektiven Erhebung aus Deutschland hatten 17 % der Patienten mit GPP eine Psoriasisarthritis, aber es wurden auch Prävalenzen von bis zu etwa einem Drittel der Patienten beschrieben.^28^ Nach gegenwärtiger Ansicht ist die GPP eine systementzündliche Multiorganerkrankung, unter anderem mit Gelenkbeteiligung.^29^ Etwa 50 % bis 60 % der Patienten mit GPP haben oder hatten auch eine Psoriasis vulgaris.^2^, ^11^, ^30^ Dabei scheint der Anteil der Patienten, bei denen zum Zeitpunkt eines Schubes der GPP eine moderate bis schwere Psoriasis vulgaris vorliegt, deutlich geringer zu sein. Des Weiteren ist die Koinzidenz mit anderen autoimmunologischen oder autoinflammatorischen Erkrankungen, insbesondere dem Morbus Crohn und der neutrophilen Cholangitis, gehäuft.^30^, ^31^, ^32^, ^33^ Die neutrophile Cholangitis ist histologisch durch eine Infiltration des Gallengangsystems durch neutrophile Granulozyten definiert und führt zu Cholestase mit Ikterus und Anstieg der typischen Cholestaseparameter (Bilirubin, Gallensäuren, Cholesterin, Gallenenzyme) sowie cholestatischem Pruritus.^31^, ^34^, ^35^, ^36^, ^37^

### Komplikationen

Epidemiologische Analysen zeigen eine erhöhte Mortalität bei Patienten mit GPP, und Todesfälle, die Schüben der GPP zugerechnet werden konnten, wurden in etwa 5 % bis 10 % der Fälle von GPP beschrieben.^4^, ^7^, ^14^, ^15^, ^38^, ^39^, ^40^ Sepsis, einzelne aseptische Pneumonitiden (acute respiratory distress syndrome) sowie Augenentzündungen (Konjunktivitis, Uveitis oder Iritis) wurden als schwere Komplikationen beschrieben.^14^, ^38^, ^41^, ^42^, ^43^, ^44^, ^45^, ^46^ Bei schweren erythrodermatischen Verläufen ist insbesondere das Infektionsrisiko durch Bakterien erhöht. Außerdem kann es durch Albuminverlust und hepatische Syntheseinsuffizienz zu Hypalbuminämie und Ödemen kommen.^47^ In Einzelfällen werden auch Veränderungen des Kalziumstoffwechsels im Sinne eines primären Hypoparathyreoidismus beschrieben. Dabei ist unklar, ob er als Auslöser oder Folge der GPP zu werten ist.^48^, ^49^, ^50^, ^51^

### Klinische Bewertungsmaßstäbe (Scores)

Der Schweregrad der GPP wird in Anlehnung an Bewertungsmaßstäbe der Psoriasis vulgaris durch den *Generalized Pustular Psoriasis Area and Severity Index* (GPPASI) und das *Generalized Pustular Psoriasis Physician Global Assessment* (GPPGA) gemessen.^52^, ^53^ Der GPPASI entspricht dem PASI mit Gesamtwerten von 0 bis 72, wobei das Kriterium „Induration“ durch „Pusteln“ ersetzt wird. Der GPPGA bewertet den Schweregrad der GPP anhand eines *Physician Global Assessment* (PGA). Pusteln, Erythem und Schuppung werden bewertet, wobei jede Komponente Werte von 0 bis 4 erreichen kann.^9^

Der JDASI (*Japanese Dermatological Association Severity Index*) bezieht neben Hautveränderungen auch systemische Parameter ein. Es werden maximal 9 Punkte für den Hautbefund (Erythemfläche gesamt, Erythemfläche mit Pusteln, Ödemfläche) sowie 8 Punkte für systemische Beteiligung (Pyrexie, Leukozytenzahl, CRP, Serumalbumin) vergeben. Somit wird ein Score von 0 bis 17 Punkten errechnet (Schweregrad: 0 bis 6 Punkte: leicht, 7 bis 10 Punkte: mittelschwer, 11 bis 17 Punkte: schwer).^47^

### Diagnostik

Empfehlungen zur Diagnostik sind in Tabelle 2 dargestellt.

**TABELLE 2:** Empfehlungen zur Diagnostik.

Empfehlung
Die Eruierung möglicher Provokationsfaktoren wird empfohlen.
Zur Beurteilung des Gesamtzustandes der Patienten werden Laboruntersuchungen (Blutbild inklusive Differenzialblutbild, Nierenretentionsparameter, Leberfunktionsparameter, Serum‐Elektrolyte und CRP) empfohlen.
Die Bestimmung von Albumin, Kalzium und Procalcitonin im Serum kann bei reduziertem Allgemeinzustand empfohlen werden.
Die histologische Untersuchung eines Hautbiopsats wird empfohlen. Eine PAS‐Färbung sollte zum Ausschluss einer Mykose herangezogen werden.
Zur Schweregradeinteilung kann die Verwendung validierter Instrumente (GPPASI, GPPGA, JDASI) empfohlen werden.
Zur Evaluation einer AGEP als Differenzialdiagnose wird das Heranziehen der Kriterien der EuroSCAR‐Studiengruppe empfohlen.
Ein Screening auf Komorbidität (kardiovaskuläre, metabolische, psychische und chronisch‐entzündliche Erkrankungen) kann empfohlen werden.

*Abk*.: AGEP, akute generalisierte exanthematische Pustulose; CRP, C‐reaktives Protein; GPPGA, *Generalized Pustular Psoriasis Physician Global Assessment*; GPPASI, *Generalized Pustular Psoriasis Area and Severity Index*; JDASI, *Japanese Dermatological Association Severity Index*; PAS *Periodic acid‐Schiff*

### Differenzialdiagnosen

In Abbildung 3 werden differenzialdiagnostische Kriterien dargestellt. Siehe auch Langfassung der Leitlinie

### Versorgungslage

Siehe Langfassung der Leitlinie.

### Therapie

#### Einleitung Therapie

Zur Therapie der GPP werden verschiedene Modalitäten angewandt, von denen manche auch kombiniert werden können. Dazu zählen topische Therapien, UV‐Therapien, medikamentöse Systemtherapien und die in Europa kaum angewandte Granulozyten‐/Monozyten‐Adsorptionsapherese (GMA). Während bei weniger ausgeprägten Formen topische Therapie und UV‐Therapien ausreichen können, sind bei schwereren Formen oder bei therapieresistenten Verläufen weitere Systemtherapien erforderlich. Wenn eine im akuten Schub eingeleitete medikamentöse Systemtherapie als erfolgreich bewertet wird, sollte dieser Wirkstoff in der Erhaltungstherapie vorrangig beibehalten werden. Dies gilt nicht für systemische Glukokortikoide. Die Applikationsart (i.v. versus s.c.) bei Spesolimab soll gemäß den Zulassungen erfolgen. Bei Kombinationstherapien müssen Interaktionen und Kontraindikationen berücksichtigt werden. Spezifische Aspekte zur Therapie in der Schwangerschaft werden in einem eigenen Abschnitt behandelt.

Primär werden Evidenzen der Therapien bei GPP beleuchtet. Für die Geschwindigkeit des Wirkeintritts werden vorliegende Studiendaten sowie eigene Erfahrungen der Autoren bei der GPP berücksichtigt. Ferner fließen Erfahrungen aus anderen Indikationen wie bei der Behandlung der Psoriasis vulgaris ein. Zu Anwendungshinweisen, Laborkontrollen, Arzneimittelinteraktionen, Kontraindikationen sowie besonderen Aspekten während der Behandlung wird auf Fachinformationen und Leitlinien zu anderen Indikationen verwiesen. Für bei der Psoriasis vulgaris zugelassene Therapien werden praktische Aspekte zum Einsatz ausführlich in der aktuellen S3‐Leitlinie „Therapie der Psoriasis“ behandelt.^54^, ^55^ Wir empfehlen die Orientierung an Dosierungen bei Psoriasis sofern dafür zugelassen und im Folgenden nicht anders spezifiziert. Eine deutsche Leitlinie oder stratifizierte Behandlungsempfehlung zur GPP existierte bislang nicht. In Japan wurde 2018 eine Leitlinie zur GPP veröffentlicht, die in die zugrunde liegende Datenauswertung vor allem asiatische Patienten einbezogen hatte.^27^ Manche Patienten mit GPP leiden gleichzeitig an Psoriasis vulgaris oder Psoriasisarthritis, was bei der Wahl des Systemtherapeutikums berücksichtigt werden sollte.

**Table:** 

Statement
Die GPP ist gemäß der Beurteilung der Autoren der vorliegenden Leitlinie eine seltene, schwerwiegende und potenziell letal verlaufende Erkrankung, die die Lebensqualität der Betroffenen stark einschränkt. Im Zuge der herausfordernden Behandlung muss häufig auf Therapien zurückgegriffen werden, für die keine Zulassung für die GPP vorliegt (*off‐label use*) und für die oft auch keine kontrollierten Studien vorhanden sind. Bei Vorliegen entsprechender Begleiterkrankungen wie insbesondere einer Psoriasis vulgaris oder Psoriasisarthritis, können die in dieser Leitlinie diskutierten Therapeutika teils im Rahmen der Zulassung dieser Erkrankungen eingesetzt werden.

#### Topische Therapie

Empfehlung 4.2.1‐4.2.3 der AWMF‐Leitlinie

**Table:** 

Wir empfehlen im akuten Schub einer GPP Glukokortikoide der Klasse IV (Clobetasol). Aufgrund ihres ungünstigen therapeutischen Indexes sollten sie möglichst nur kurzfristig zur Therapie eines akuten Schubs, insbesondere auch bis zum Wirkeintritt systemischer Therapien, verwendet werden. Bei leichten Schüben sowie bei mittel‐ und längerfristiger Therapie empfehlen wir, topische Glukokortikoide der Klassen II oder III mit hohem therapeutischen Index wie Prednicarbat oder Mometasonfuroat zu verwenden.^56^ Eine langfristige großflächige Anwendung topischer Glukokortikoide, insbesondere als Monotherapie, sollte möglichst vermieden werden.
Die topische Therapie mit Vitamin‐D3‐Analoga (*off‐label*) wird bei akuter GPP nicht empfohlen. Ihr Einsatz, auch als Fixkombination mit einem Glukokortikoid (*off‐label*), kann zur Erhaltungstherapie und bei geringerer Krankheitslast der GPP erwogen werden.
Topisches Tacrolimus (*off‐label*) kann in Einzelfällen zur mittel‐ oder längerfristigen Therapie bei GPP erwogen werden, zum Beispiel bei Patienten mit Steroidphobie oder bei Befall sensitiver Areale wie Gesicht und Genitalien.

Für Hintergrundinformationen verweisen wir auf die Langfassung der Leitlinie.

#### Physikalische Therapie

Empfehlung 4.3.1.‐4.3.2 der AWMF‐Leitlinie

**Table:** 

Die Psoralen‐UVA (PUVA)‐Therapie kann bei GPP erwogen werden, wegen des eher langsamen Wirkbeginns vor allem zur Stabilisierung eines Behandlungserfolgs. Sie kann mit anderen systemischen Therapien kombiniert werden, wobei die gemeinsame Gabe mit Ciclosporin kontraindiziert ist. Die Bade‐PUVA kann in Hinblick auf das Nebenwirkungsprofil und die Praktikabilität für den Patienten gegenüber der oralen PUVA von Vorteil sein.
Die UVB‐Lichttherapie kann vor allem zur Stabilisierung eines Behandlungserfolgs empfohlen werden, als Kombinationstherapie, wenn die Patienten auf die systemische Therapie nicht ausreichend ansprechen, oder wenn eine systemische Therapie vom Patienten nicht gewünscht wird.
Die Granulozyten‐/Monozyten‐Adsorptionsapherese (GMA) kann empfohlen werden, wenn systemische Therapien nicht ausreichend wirken oder kontraindiziert sind.

Für Hintergrundinformationen verweisen wir auf die Langfassung der Leitlinie.

#### Systemtherapie

Für Hintergrundinformationen und eine Zusammenstellung der prospektiven Arzneimittelstudien verweisen wir auf die Langfassung der Leitlinie.

### Systemische Glukokortikoide

Empfehlung 4.4.1.1. der AWMF‐Leitlinie

**Table:** 

Es wird empfohlen, systemische Glukokortikoide bei der GPP zurückhaltend einzusetzen. Sie können kurzfristig im akuten Schub, insbesondere zur Überbrückung bis zum Einsetzen der Wirkung anderer Therapien, gegeben werden. Ihre Anwendung sollte möglichst in Kombination mit anderen Systemtherapeutika oder UV‐Lichttherapie erfolgen, auch um die Wahrscheinlichkeit für Schübe nach dem Absetzen zu verringern. Nach Erfahrung der Autoren kann allerdings zur kurzfristigen Überbrückung bei manchen Patienten auch die topische Behandlung mit Glukokortikoiden der Klasse IV ausreichen. Der Einsatz systemischer Glukokortikoide zur Erhaltungstherapie bei GPP wird nicht empfohlen.

### Retinoide

Empfehlung 4.4.1.2 der AWMF‐Leitlinie

**Table:** 

Acitretin wird zur Erhaltungstherapie bei Patienten mit GPP empfohlen. Für die Schubtherapie kann Acitretin als Therapie der zweiten Wahl empfohlen werden, da bei schweren akuten Formen schneller wirksame Medikamente vorgezogen werden sollten. Bei Frauen im gebärfähigen Alter sollten aufgrund der Teratogenität der Retinoide andere Therapien bevorzugt eingesetzt werden.
Etretinat wird nicht zur Therapie der GPP empfohlen, da Acitretin sich als ein alternatives Retinoid mit kürzerer Halbwertszeit in Deutschland durchgesetzt hat. Etretinat wird in Deutschland nicht mehr vermarktet.
Isotretinoin kann zur Therapie der GPP als Therapie der 3. Wahl erwogen werden, sofern Acitretin aus substanzspezifischen Gründen, wie der auch nach Absetzen langanhaltenden teratogenen Wirkung, nicht infrage kommt.

### Ciclosporin

Empfehlung 4.4.1.3 der AWMF‐Leitlinie

**Table:** 

Die Therapie mit Ciclosporin (CsA) kann im akuten Schub als zweite Wahl empfohlen werden und zur Erhaltungstherapie kann sie ebenfalls als zweite Wahl zeitlich begrenzt empfohlen werden.

### Methotrexat

Empfehlung 4.4.1.4 der AWMF‐Leitlinie

**Table:** 

Methotrexat (MTX) wirkt bei GPP bei bekanntem Sicherheitsprofil. Es kann als Therapie der zweiten Wahl zur Erhaltungstherapie empfohlen werden. Zur Therapie eines GPP‐Schubes kann die Gabe von MTX (*off‐label*) erwogen werden.

### Apremilast, Dapson und Mycophenolatmofetil

Empfehlung 4.4.1.7 der AWMF‐Leitlinie

**Table:** 

Der Inhibitor der Phosphodiesterase 4 (PDE4) Apremilast (*off‐label*) oder Dapson können bei GPP als Therapeutika der dritten Wahl erwogen werden. Mycophenolatmofetil (*off‐label*) kann nicht empfohlen werden.

### Biologika

*Tumornekrosefaktor (TNF)‐Inhibitoren*

Empfehlung 4.4.2.1 der AWMF‐Leitlinie

**Table:** 

Für den akuten Schub der GPP kann der TNF‐Inhibitor Adalimumab (*off‐label*) empfohlen werden, aufgrund des langsameren Wirkeintritts im Vergleich zu Infliximab i.v. als Mittel der zweiten Wahl. Zur Erhaltungstherapie wird Adalimumab (*off‐label*) als eine Therapie der ersten Wahl empfohlen. Bei unzureichender Wirksamkeit kann die Dosis auf 80 mg alle 2 Wochen oder 40 mg wöchentlich s.c. gesteigert werden.
Der TNF‐Inhibitor Certolizumab (*off‐label*) wird zur Erhaltungstherapie bei GPP als erste Wahl empfohlen. Bei unzureichender Wirksamkeit der 200‐mg‐Dosis kann die Steigerung auf 400 mg alle 2 Wochen erfolgen. Beim akuten Schub der GPP kann Certolizumab (off‐label) empfohlen werden, aufgrund des langsameren Wirkeintritts im Vergleich zu Infliximab i.v. als Therapeutikum der zweiten Wahl. Eine wichtige Rolle spielt das Medikament bei schwangeren Patientinnen.
Der TNF‐Inhibitor Etanercept (*off‐label*) wird zur Erhaltungstherapie der GPP als Medikament der ersten Wahl empfohlen. Im akuten Schub kann es als Therapie der zweiten Wahl (*off‐label*) empfohlen werden.
Der TNF‐Inhibitor Infliximab (*off‐label*) wird sowohl für den akuten Schub der GPP als auch zur Erhaltungstherapie als erste Wahl empfohlen. Es wird empfohlen, beim Schub der GPP Infliximab i.v. zu behandeln aufgrund des schnellen Ansprechens und der gewichtsadaptierten Dosierung. Die Umstellung einer begonnenen intravenösen Infliximabgabe auf subkutane Applikation kann bei stabil gutem Befund empfohlen werden.

*Ustekinumab*

Empfehlung 4.4.2.2 der AWMF‐Leitlinie

**Table:** 

Der Interleukin (IL)‐12 und IL‐23‐Antagonist Ustekinumab *(off‐label*) kann als Therapie der zweiten Wahl des akuten Schubs sowie als Erhaltungstherapie der GPP empfohlen werden.

*IL‐17‐ und IL‐17R‐Antagonisten*

Empfehlung 4.4.2.3 der AWMF‐Leitlinie

**Table:** 

Die IL‐17A‐Antagonisten Secukinumab (*off‐label*) und Ixekizumab (*off‐label*) werden sowohl für die Behandlung eines Schubes der GPP als auch in der Erhaltungstherapie empfohlen. Der IL‐17‐Rezeptorantagonist Brodalumab kann sowohl für die Behandlung eines Schubes der GPP als auch in der Erhaltungstherapie empfohlen werden. Der IL‐17A und F‐Antagonist Bimekizumab kann sowohl für die Behandlung eines Schubes der GPP als auch in der Erhaltungstherapie der GPP empfohlen werden.

*IL‐23‐Antagonisten*

Empfehlung 4.4.2.4 der AWMF‐Leitlinie

**Table:** 

Die IL‐23‐Antagonisten Risankizumab (*off‐label*) und Guselkumab (*off‐label*) werden sowohl zur Behandlung von GPP‐Schüben als auch zur Erhaltungstherapie empfohlen. Der IL‐23‐Antagonist Tildrakizumab (*off‐label*) kann zur Behandlung von GPP‐Schüben als auch zur Erhaltungstherapie erwogen werden.

*IL‐1‐Inhibitoren*

Empfehlung 4.4.2.5 der AWMF‐Leitlinie

**Table:** 

Die Therapie der GPP mit dem IL‐1‐Rezeptorantagonist Anakinra (*off‐label*) oder Antikörper gegen IL‐1β Canakinumab (*off‐label*) kann als Therapie der dritten Wahl erwogen werden, das heißt, sofern Therapien der ersten oder zweiten Wahl erfolglos waren, nicht durchgeführt werden können oder keinen ausreichenden Erfolg erwarten lassen. Eine Therapie mit dem IL‐1β‐Inhibitor Gevokizumab kann nicht empfohlen werden, da es von der *European Medicines Agency* (EMA) nicht zugelassen ist und kein Vorteil gegenüber den für andere Indikationen zugelassenen Biologika Anakinra und Canakinumab ersichtlich ist.

*IL‐36‐Rezeptor‐Blocker*

Empfehlung 4.4.2.6 der AWMF‐Leitlinie

**Table:** 

Der IL‐36‐Rezeptor‐Antikörper Spesolimab i.v. wird aufgrund seiner Zulassung in dieser Indikation zur Behandlung von Schüben der GPP als Therapie der ersten Wahl empfohlen. Bei weiteren Schüben wird eine erneute Gabe von Spesolimab i.v. empfohlen. Im Falle eines Therapieansprechens auf die i.v.‐Gabe(n) wird die Umstellung auf Spesolimab s.c. als Erhaltungstherapie empfohlen. Spesolimab s.c. wird zur Vorbeugung von Schüben der GPP aufgrund seiner Zulassung in dieser Indikation als Therapie der ersten Wahl empfohlen. Das heißt, dass der Einsatz von Spesolimab s.c. nach Schüben einer GPP bei dann fehlender oder niedriger klinischer Aktivität der GPP empfohlen wird. Nach Rückzug von Spesolimab i.v. vom Markt in Deutschland kann Spesolimab i.v. nur über die Auslandsapotheke bezogen werden. Die Kostenübernahme sollte vorab mit dem Kostenträger geklärt werden. Bei Spesolimab s.c. empfehlen wir, falls zukünftig keine Marktverfügbarkeit in der BRD bestehen sollte, ein ähnliches Vorgehen.

### Therapie in der Schwangerschaft

#### Empfehlung 4.5 der AWMF‐Leitlinie

Bei schwerem Verlauf in der Schwangerschaft empfehlen wir in erster Linie Certolizumab s.c. als Therapie der zweiten Wahl kann der Einsatz von Adalimumab oder Infliximab empfohlen werden. Eine Therapie mit Ustekinumab kann erwogen werden.

Prednisolon kann ergänzend zur Überbrückung insbesondere bei schweren Verläufen erwogen werden, eignet sich jedoch nicht für eine Langzeittherapie. Eine Therapie mit CsA kann grundsätzlich ebenfalls erwogen werden. CsA verfügt jedoch über ein schlechteres Sicherheitsprofil als die genannten Alternativen. Acitretin und MTX sind in der Schwangerschaft absolut kontraindiziert und ihr Einsatz wird nicht empfohlen. Bei milden Verläufen empfehlen wir die topische Anwendung von Glukokortikoiden. Ebenso kann die UVB‐Therapie bei weniger ausgeprägtem Hautbefund oder zur Überbrückung empfohlen werden. Bei schweren Verläufen kann diese Therapie zusätzlich zu Biologika empfohlen werden. Eine engmaschige interdisziplinäre ärztliche Kontrolle der Schwangeren wird empfohlen.

Für Hintergrundinformationen verweisen wir auf die Langfassung der Leitlinie.

## DANKSAGUNG

Open access Veröffentlichung ermöglicht und organisiert durch Projekt DEAL.

## INTERESSENKONFLIKT

Eine tabellarische Auflistung der Interessenkonflikte aller beteiligten Parteien ist in der Langfassung der Leitlinie zu finden.
